# Supplementary figures and images for: Improved inter-subject alignment of the lumbosacral cord for group-level in vivo gray and white matter assessments: A scan-rescan MRI study at 3T
Source: PLoS One. 2024 Apr 16;19(4):e0301449. doi: 10.1371/journal.pone.0301449 (PMC11020367; doi:10.1371/journal.pone.0301449)

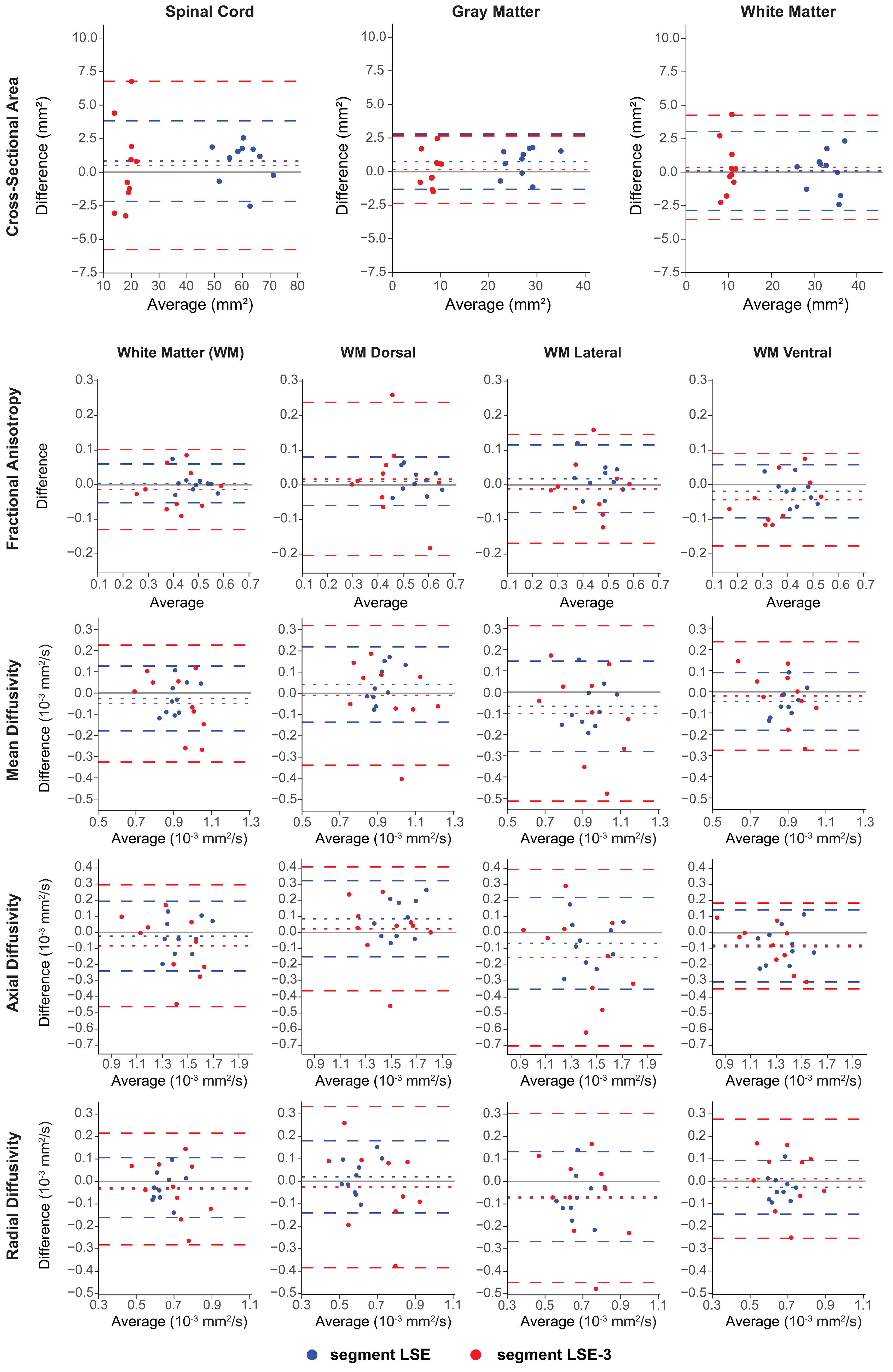

Supplement: S1 Fig — The dotted lines represent the bias, i.e., the mean of the scan-rescan differences, while the dashed lines represent the 95% limits of agreement. (TIF) [file pone.0301449.s001.tif]
