# Supplementary material for: Improved inter-subject alignment of the lumbosacral cord for group-level in vivo gray and white matter assessments: A scan-rescan MRI study at 3T
Source: PLoS One. 2024 Apr 16;19(4):e0301449. doi: 10.1371/journal.pone.0301449 (PMC11020367; doi:10.1371/journal.pone.0301449)
Supplement: S1 Table — (DOCX) [file pone.0301449.s002.docx]

**S1 Table.** Demographic and clinical information of patients with spinal cord injury.

| ***ID*** | ***Sex*** | ***Age (y)*** | ***Time since injury (m)*** | ***Neurological level of injury*** | ***AIS*** | ***Type of injury*** | ***Aetiology*** |
| --- | --- | --- | --- | --- | --- | --- | --- |
| 1 | M | 59 | 6.1 | C1 | D | Traumatic | Dislocation fracture (C1) |
| 2 | F | 21 | 5.8 | C4 | D | Traumatic | Dislocation fracture (C4-C5) |
| 3 | M | 64 | 5.9 | C6 | D | Traumatic | Dislocation fracture (C5-C7) |
| 4 | M | 58 | 5.8 | T2 | B | Non-traumatic | Ischaemic (T3) |
| 5 | M | 26 | 5.8 | T10 | D | Non-traumatic | Acute myelitis |

*Abbreviations:* AIS, American Spinal Injury Association Impairment Scale; F, female; M, male.
