# Supplementary material for: Improved inter-subject alignment of the lumbosacral cord for group-level in vivo gray and white matter assessments: A scan-rescan MRI study at 3T
Source: PLoS One. 2024 Apr 16;19(4):e0301449. doi: 10.1371/journal.pone.0301449 (PMC11020367; doi:10.1371/journal.pone.0301449)
Supplement: S2 Table — (DOCX) [file pone.0301449.s003.docx]

**S2 Table.** Slice-wise cross-sectional area values when aligning the individual slice stacks at different lumbosacral enlargement landmarks.

|  | Distance from  LSE landmark (mm) | Cross-sectional Area (mm^2^) | | |
| --- | --- | --- | --- | --- |
|  |  | Spinal Cord | Gray Matter | White Matter |
| LSE landmark: $\mathrm{SC}_{max,mw}$ | +10 | 59.4 ± 5.3 | 21.0 ± 3.3 | 38.5 ± 2.9 |
|  | +5 | 62.2 ± 6.6 | 23.5 ± 3.7 | 38.6 ± 3.9 |
|  | 0 | 63.0 ± 5.6 | 25.0 ± 3.5 | 38.0 ± 3.3 |
|  | -5 | 62.0 ± 5.6 | 26.8 ± 4.6 | 35.2 ± 2.9 |
|  | -10 | 59.3 ± 5.8 | 27.8 ± 3.8 | 31.5 ± 4.0 |
|  | -15 | 53.2 ± 6.5 | 24.7 ± 3.7 | 28.5 ± 3.8 |
|  | -20 | 43.3 ± 8.5 | 20.0 ± 4.3 | 23.3 ± 4.7 |
|  | -25 | 32.1 ± 9.4 | 14.7 ± 4.7 | 17.4 ± 4.8 |
|  | -30 | 22.4 ± 7.1 | 10.0 ± 3.4 | 12.4 ± 3.8 |
|  | -35 | 15.3 ± 6.6 | 6.6 ± 3.3 | 8.7 ± 3.4 |
|  | -40 | 10.9 ± 5.5 | 4.1 ± 2.8 | 6.8 ± 2.8 |
| LSE landmark: $\mathrm{GM}_{max,mw}$ | +20 | 57.3 ± 4.9 | 19.5 ± 3.2 | 37.8 ± 2.7 |
|  | +15 | 60.6 ± 5.3 | 22.1 ± 2.9 | 38.6 ± 3.3 |
|  | +10 | 62.2 ± 6.2 | 23.7 ± 3.3 | 38.5 ± 3.8 |
|  | +5 | 62.2 ± 5.5 | 26.2 ± 2.9 | 36.0 ± 3.1 |
|  | 0 | 60.8 ± 6.8 | 27.8 ± 4.1 | 33.0 ± 4.3 |
|  | -5 | 56.3 ± 7.1 | 27.3 ± 4.6 | 29.0 ± 3.2 |
|  | -10 | 48.7 ± 7.8 | 22.4 ± 3.5 | 26.2 ± 4.5 |
|  | -15 | 35.5 ± 7.5 | 16.6 ± 3.8 | 18.9 ± 4.1 |
|  | -20 | 24.9 ± 6.8 | 11.2 ± 2.8 | 13.8 ± 4.2 |
|  | -25 | 17.4 ± 5.5 | 7.4 ± 2.6 | 10.0 ± 3.1 |
|  | -30 | 12.1 ± 4.2 | 4.9 ± 2.1 | 7.2 ± 2.1 |

*Notes:* Values represent mean ± standard deviation across 10 healthy volunteers. A positive distance indicates a rostral direction from the LSE landmark. Values were derived from the first scan; highly comparable results were obtained from the second scan (rescan).

*Abbreviations:* GM, gray matter; LSE, lumbosacral enlargement; SC, spinal cord.
