# Supplementary material for: Improved inter-subject alignment of the lumbosacral cord for group-level in vivo gray and white matter assessments: A scan-rescan MRI study at 3T
Source: PLoS One. 2024 Apr 16;19(4):e0301449. doi: 10.1371/journal.pone.0301449 (PMC11020367; doi:10.1371/journal.pone.0301449)
Supplement: S3 Table — (DOCX) [file pone.0301449.s004.docx]

**S3 Table**. Slice-wise intra- and inter-rater reliability of cross-sectional area measurements (n=10; 5 healthy controls and 5 patients with spinal cord injury).

|  | Distance from LSE landmark (mm) | Intra-rater CV (%) | | | Inter-rater CV (%) | | |
| --- | --- | --- | --- | --- | --- | --- | --- |
|  |  | Controls | Patients | Diff. | Controls | Patients | Diff. |
| Spinal Cord | +20 | 2.3 | 2.5 | 0.2 | 4.4 | 5.5 | 1.2 |
|  | +15 | 1.6 | 2.1 | 0.5 | 4.4 | 4.6 | 0.2 |
|  | +10 | 2.1 | 2.2 | 0.2 | 3.7 | 4.6 | 0.9 |
|  | +5 | 2.2 | 2.4 | 0.2 | 3.2 | 3.7 | 0.5 |
|  | 0 | 1.6 | 2.7 | 1.1 | 4.7 | 5.4 | 0.7 |
|  | -5 | 2.0 | 2.9 | 0.9 | 5.6 | 6.4 | 0.8 |
|  | -10 | 3.0 | 2.6 | -0.4 | 6.9 | 11.1 | 4.2 |
|  | -15 | 3.8 | 3.7 | -0.1 | 6.1 | 12.0 | 6.0 |
|  | -20 | 4.0 | 4.0 | 0.0 | 4.9 | 13.7 | 8.7 |
|  | -25 | 4.5 | 6.3 | 1.7 | 5.6 | 21.0 | 15.3 |
|  | -30 | 8.3 | 7.2 | -1.0 | 12.0 | 20.4 | 8.5 |
| Gray Matter | +20 | 3.8 | 4.0 | 0.2 | 6.1 | 9.1 | 3.0 |
|  | +15 | 5.0 | 4.6 | -0.4 | 3.7 | 8.4 | 4.7 |
|  | +10 | 4.2 | 3.5 | -0.7 | 5.4 | 6.9 | 1.5 |
|  | +5 | 3.8 | 3.3 | -0.5 | 3.7 | 6.0 | 2.3 |
|  | 0 | 4.0 | 3.3 | -0.7 | 6.1 | 7.5 | 1.5 |
|  | -5 | 2.6 | 2.9 | 0.3 | 5.0 | 9.6 | 4.6 |
|  | -10 | 3.5 | 4.3 | 0.8 | 6.1 | 14.8 | 8.7 |
|  | -15 | 3.3 | 4.7 | 1.4 | 7.3 | 16.5 | 9.2 |
|  | -20 | 5.0 | 6.9 | 2.0 | 7.5 | 18.6 | 11.1 |
|  | -25 | 6.3 | 8.3 | 2.0 | 10.4 | 17.9 | 7.5 |
|  | -30 | 10.8 | 10.2 | -0.6 | 13.5 | 14.3 | 0.9 |
| White Matter | +20 | 3.0 | 2.9 | 0.0 | 6.6 | 7.3 | 0.8 |
|  | +15 | 3.2 | 3.2 | -0.1 | 6.0 | 6.7 | 0.7 |
|  | +10 | 3.3 | 4.1 | 0.8 | 6.1 | 5.5 | -0.7 |
|  | +5 | 3.5 | 3.6 | 0.1 | 5.2 | 4.0 | -1.2 |
|  | 0 | 1.9 | 4.1 | 2.2 | 4.5 | 5.5 | 1.0 |
|  | -5 | 2.5 | 4.4 | 1.9 | 6.9 | 5.4 | -1.6 |
|  | -10 | 5.3 | 4.9 | -0.5 | 10.4 | 10.5 | 0.1 |
|  | -15 | 5.6 | 5.7 | 0.1 | 11.1 | 11.0 | -0.1 |
|  | -20 | 5.8 | 6.5 | 0.7 | 8.9 | 10.9 | 2.0 |
|  | -25 | 5.3 | 9.7 | 4.4 | 13.3 | 25.1 | 11.8 |
|  | -30 | 10.0 | 9.6 | -0.4 | 18.0 | 27.9 | 10.0 |

*Notes:* CSA values represent an average across values obtained by three raters, based on the first set of segmentation of each rater. Differences represent patients vs. controls comparisons. The individual axial slice stacks were aligned at the LSE landmark, defined as the slice with the largest gray matter CSA ($\mathrm{GM}_{max,mw}$), without adjustment for the length of the conus medullaris. A positive distance indicates a rostral direction from the LSE landmark. For a single subject, GM and WM CSA values were not available for slices with coordinates +20 and -30 mm (n=9).

*Abbreviations:* CV, coefficient of variation; CSA, cross-sectional area; LSE, lumbosacral enlargement.
