# Supplementary material for: Improved inter-subject alignment of the lumbosacral cord for group-level in vivo gray and white matter assessments: A scan-rescan MRI study at 3T
Source: PLoS One. 2024 Apr 16;19(4):e0301449. doi: 10.1371/journal.pone.0301449 (PMC11020367; doi:10.1371/journal.pone.0301449)
Supplement: S4 Table — (DOCX) [file pone.0301449.s005.docx]

**S5 Table**. Slice-wise scan-rescan reliability of cross-sectional area measurements (n=10 healthy volunteers).

|  | Distance from LSE landmark (mm) | CSA (mm^2^)  mean ± SD | $\bar{d}$ (mm^2^)  [± 1.96 SD] | CV  (%) | ICC  [±95% CI] | MDC  (%) |
| --- | --- | --- | --- | --- | --- | --- |
| Spinal Cord | +20 | 57.5 ± 5.2 | -0.5 [±5.3] | 2.9 | .88 [.60, .97] | 8.7 |
|  | +15 | 60.9 ± 5.7 | -0.5 [±3.0] | 1.5 | .96 [.87, .99] | 4.9 |
|  | +10 | 62.1 ± 5.7 | 0.2 [±3.4] | 1.6 | .96 [.84, .99] | 5.2 |
|  | +5 | 61.9 ± 5.8 | 0.5 [±2.7] | 1.4 | .97 [.89, .99] | 4.3 |
|  | 0 | 60.4 ± 6.9 | 0.7 [±3.9] | 2.1 | .96 [.85, .99] | 6.4 |
|  | -5 | 55.7 ± 7.0 | 1.2 [±3.8] | 2.3 | .95 [.79, .99] | 7.6 |
|  | -10 | 47.4 ± 7.7 | 2.5 [±8.9] | 7.0 | .81 [.41, .95] | 20.1 |
|  | -15 | 34.9 ± 7.9 | 1.2 [±5.7] | 5.3 | .93 [.75, .98] | 16.5 |
|  | -20 | 24.6 ± 6.7 | 0.6 [±7.6] | 9.7 | .85 [.52, .96] | 29.1 |
|  | -25 | 17.2 ± 5.7 | 0.5 [±6.5] | 11.7 | .86 [.53, .96] | 35.5 |
|  | -30 | 11.8 ± 4.4 | 0.5 [±7.1] | 18.6 | .70 [.11, .93] | 60.0 |
| Gray Matter | +20 | 19.8 ± 3.1 | -0.6 [±3.9] | 4.8 | .81 [.42, .95] | 19.4 |
|  | +15 | 22.3 ± 2.7 | -0.5 [±2.6] | 3.6 | .88 [.61, .97] | 11.7 |
|  | +10 | 23.9 ± 3.1 | -0.5 [±2.7] | 3.6 | .91 [.70, .98] | 10.9 |
|  | +5 | 26.1 ± 3.0 | 0.3 [±2.7] | 3.2 | .90 [.68, .98] | 10.0 |
|  | 0 | 27.5 ± 3.9 | 0.5 [±2.7] | 3.4 | .93 [.77, .98] | 9.9 |
|  | -5 | 26.5 ± 4.2 | 1.7 [±4.9] | 6.8 | .79 [.32, .94] | 20.9 |
|  | -10 | 21.8 ± 3.8 | 1.1 [±3.9] | 6.6 | .85 [.50, .96] | 19.2 |
|  | -15 | 16.3 ± 3.9 | 0.5 [±3.3] | 7.0 | .91 [.70, 98] | 19.7 |
|  | -20 | 11.3 ± 2.9 | -0.3 [±3.3] | 8.7 | .86 [.54, .96] | 27.3 |
|  | -25 | 7.3 ± 2.5 | 0.2 [±2.8] | 10.5 | .86 [.55, .96] | 35.8 |
|  | -30 | 4.9 ± 2.3 | 0.0 [±3.2] | 18.5 | .78 [.27, .95] | 63.9 |
| White Matter | +20 | 37.7 ± 2.6 | 0.2 [±4.4] | 3.4 | .70 [.14, .92] | 10.9 |
|  | +15 | 38.6 ± 3.6 | 0.0 [±2.9] | 2.1 | .92 [.72, .98] | 7.0 |
|  | +10 | 38.2 ± 3.2 | 0.7 [±3.6] | 2.6 | .84 [.51, .96] | 9.4 |
|  | +5 | 35.9 ± 3.3 | 0.2 [±2.3] | 2.1 | .94 [.79, .99] | 6.0 |
|  | 0 | 32.9 ± 4.1 | 0.2 [±3.9] | 3.4 | .90 [.64, .97] | 11.1 |
|  | -5 | 29.2 ± 3.1 | -0.5 [±4.1] | 4.1 | .81 [.41, .95] | 13.3 |
|  | -10 | 25.5 ± 4.1 | 1.4 [±7.1] | 8.8 | .66 [.15, .90] | 28.1 |
|  | -15 | 18.6 ± 4.1 | 0.6 [±3.3] | 5.5 | .92 [.71, .98] | 17.8 |
|  | -20 | 13.3 ± 3.8 | 0.9 [±5.1] | 12.2 | .79 [.39, .94] | 37.7 |
|  | -25 | 9.9 ± 3.3 | 0.3 [±3.9] | 12.5 | .85 [.50, .96] | 36.7 |
|  | -30 | 6.9 ± 2.1 | 0.5 [±4.3] | 19.2 | .56 [-.13, .88] | 63.4 |

*Notes:* The individual axial slice stacks were aligned at the LSE landmark, defined as the slice with the largest gray matter CSA ($\mathrm{GM}_{max,mw}$), without adjusting for the length of the conus medullaris. The landmarks were determined in the first scan. A positive distance indicates a rostral direction from the LSE landmark. For a single subject, CSA values were not available for slices with coordinates -30 mm (n=9).

*Abbreviations:* CI, confidence interval; CV, scan-rescan coefficient of variation; CSA, cross-sectional area; $\bar{d}$, mean scan-rescan difference; ICC, scan-rescan intraclass correlation coefficient; LSE, lumbosacral enlargement; MDC, minimal detectable change; SD, standard deviation.
