# Supplementary material for: Improved inter-subject alignment of the lumbosacral cord for group-level in vivo gray and white matter assessments: A scan-rescan MRI study at 3T
Source: PLoS One. 2024 Apr 16;19(4):e0301449. doi: 10.1371/journal.pone.0301449 (PMC11020367; doi:10.1371/journal.pone.0301449)
Supplement: S5 Table — (DOCX) [file pone.0301449.s006.docx]

**S6 Table.** Slice-wise scan-rescan reliability of fractional anisotropy and mean diffusivity values (n=10 healthy volunteers).

|  | Distance from LSE landmark (mm) | **Fractional Anisotropy** | | | | | **Mean Diffusivity** (10^-3^ mm^2^/s) | | | | |
| --- | --- | --- | --- | --- | --- | --- | --- | --- | --- | --- | --- |
|  |  | mean ± SD | $\bar{d}$  [± 1.96 SD] | CV  (%) | ICC  [95% CI] | MDC  (%) | mean ± SD | $\bar{d}$  [± 1.96 SD] | CV  (%) | ICC  [95% CI] | MDC  (%) |
| Gray Matter | +20 | .41 ± .05 | .02 [±.08] | 6.3 | .72 [.24, .92] | 20.2 | .89 ± .07 | .04 [±.09]* | 4.1 | .70 [.08, .92] | 12.6 |
|  | +15 | .37 ± .05 | .01 [±.15] | 9.5 | .35 [-.37, .79] | 38.1 | .88 ± .05 | .00 [±.18] | 5.1 | .12 [-.62, .69] | 19.5 |
|  | +10 | .34 ± .05 | -.01 [±.10] | 9.2 | .60 [-.02, .88] | 28.9 | .87 ± .05 | .01 [±.09] | 2.7 | .64 [.09, .90] | 10.2 |
|  | +5 | .33 ± .05 | -.02 [±.08] | 7.3 | .72 [.25, .92] | 23.8 | .83 ± .04 | .00 [±.08] | 2.9 | .51 [-.19, .85] | 9.6 |
|  | 0 | .33 ± .04 | .01 [±.07] | 6.4 | .69 [.18, .91] | 21.9 | .81 ± .07 | -.01 [±.16] | 6.1 | .45 [-.26, .83] | 19.5 |
|  | -5 | .33 ± .06 | -.03 [±.05]* | 6.2 | .85 [.14, .97] | 20.0 | .79 ± .04 | .01 [±.18] | 6.7 | -.19 [-.82, .50] | 22.4 |
|  | -10 | .31 ± .06 | -.01 [±.07] | 6.9 | .86 [.54, .96] | 22.1 | .82 ± .03 | -.01 [±.17] | 6.1 | -.30 [-.89, .43] | 21.0 |
|  | -15 | .31 ± .06 | -.02 [±.12] | 9.9 | .60 [.02, .88] | 37.7 | .80 ± .05 | -.01 [±.12] | 3.8 | .48 [-.18, .84] | 14.8 |
|  | -20 | .33 ± .09 | .01 [±.16] | 14.7 | .65 [-.01, .91] | 45.3 | .81 ± .06 | .00 [±.20] | 6.6 | .21 [-.60, .76] | 23.5 |
| White Matter | +20 | .55 ± .03 | .00 [±.08] | 4.4 | .41 [-.32, .82] | 14.3 | .97 ± .10 | .00 [±.09] | 2.4 | .91 [.68, .98] | 8.4 |
|  | +15 | .52 ± .05 | .00 [±.13] | 5.8 | .35 [-.39, .79] | 23.6 | .97 ± .07 | -.03 [±.19] | 4.8 | .29 [-.36, .76] | 19.4 |
|  | +10 | .50 ± .07 | -.01 [±.09] | 5.4 | .80 [.37, .95] | 17.2 | .96 ± .05 | -.01 [±.09] | 2.6 | .60 [-.02, .89] | 9.1 |
|  | +5 | .48 ± .06 | .00 [±.09] | 5.4 | .77 [.31, .94] | 17.4 | .96 ± .07 | -.06 [±.14]* | 5.2 | .44 [-.10, .81] | 17.4 |
|  | 0 | .48 ± .07 | .01 [±.07] | 4.2 | .86 [.56, .96] | 14.4 | .91 ± .08 | -.03 [±.17] | 6.1 | .49 [-.11, .84] | 19.1 |
|  | -5 | .47 ± .07 | -.01 [±.06] | 3.8 | .91 [.69, .98] | 12.3 | .90 ± .06 | -.02 [±.16] | 5.7 | .41 [-.29, .81] | 17.2 |
|  | -10 | .43 ± .07 | .01 [±.11] | 7.9 | .75 [.26, .93] | 23.7 | .95 ± .10 | -.05 [±.21] | 6.1 | .56 [.00, .87] | 22.5 |
|  | -15 | .42 ± .07 | -.02 [±.10] | 7.7 | .73 [.25, .92] | 24.4 | .92 ± .09 | .04 [±.22] | 6.7 | .42 [-.19, .81] | 23.4 |
|  | -20 | .42 ± .10 | .03 [±.10] | 8.5 | .83 [.41, .96] | 26.9 | .93 ± .12 | -.07 [±.27] | 9.3 | .44 [-.17, .83] | 30.5 |
| WM Dorsal | +20 | .63 ± .04 | -.01 [±.13] | 5.8 | .31 [-.43, .78] | 19.3 | 1.00 ± .12 | .03 [±.23] | 5.6 | .60 [.00, .88] | 22.6 |
|  | +15 | .59 ± .06 | -.01 [±.17] | 7.5 | .30 [-.42, .77] | 27.2 | 1.00 ± .08 | .00 [±.21] | 5.6 | .41 [-.33, .82] | 20.3 |
|  | +10 | .59 ± .08 | -.01 [±.13] | 6.8 | .68 [.12, .91] | 21.4 | .96 ± .06 | .01 [±.15] | 4.5 | .49 [-.18, .85] | 14.5 |
|  | +5 | .56 ± .06 | .01 [±.11] | 5.9 | .71 [.16, .92] | 17.9 | .95 ± .07 | .01 [±.20] | 5.9 | .26 [-.48, .75] | 20.6 |
|  | 0 | .56 ± .07 | .02 [±.07] | 3.9 | .88 [.60, .97] | 12.7 | .92 ± .07 | .04 [±.16] | 5.6 | .50 [-.08, .84] | 18.0 |
|  | -5 | .54 ± .07 | -.01 [±.12] | 5.9 | .70 [.16, .92] | 20.3 | .89 ± .10 | .05 [±.17] | 6.6 | .61 [.08, .88] | 20.4 |
|  | -10 | .47 ± .09 | .01 [±.15] | 9.2 | .72 [.19, .92] | 29.2 | .95 ± .23 | .00 [±.26] | 7.0 | .86 [.52, .96] | 25.2 |
|  | -15 | .47 ± .07 | -.04 [±.08]* | 7.7 | .71 [.01, .93] | 22.7 | .90 ± .08 | .02 [±.26] | 8.3 | .16 [-.55, .70] | 28.6 |
|  | -20 | .45 ± .10 | .08 [±.15] | 12.6 | .59 [-.08, .89] | 42.9 | .93 ± .07 | -.06 [±.27] | 9.2 | .05 [-.50, .64] | 29.7 |
| WM Lateral | +20 | .53 ± .03 | .01 [±.08] | 4.6 | .42 [-.27, .82] | 15.1 | .95 ± .08 | -.02 [±.11] | 3.5 | .78 [.36, .94] | 11.5 |
|  | +15 | .51 ± .05 | .01 [±.15] | 6.9 | .33 [-.40, .79] | 28.0 | .95 ± .09 | -.04 [±.24] | 6.0 | .34 [-.32, .78] | 25.4 |
|  | +10 | .48 ± .06 | .00 [±.09] | 5.0 | .79 [.35, .94] | 17.2 | .97 ± .07 | -.02 [±.13] | 4.2 | .66 [.13, .90] | 13.4 |
|  | +5 | .47 ± .06 | .01 [±.14] | 8.1 | .45 [-.26, .83] | 29.1 | 1.00 ± .11 | -.14 [±.27]* | 10.0 | .27 [-.15, .70] | 34.2 |
|  | 0 | .47 ± .07 | .03 [±.13] | 8.4 | .67 [.16, .90] | 27.3 | .93 ± .12 | -.08 [±.28] | 9.7 | .45 [-.11, .82] | 31.6 |
|  | -5 | .45 ± .07 | .00 [±.06] | 4.5 | .92 [.70, .98] | 13.2 | .92 ± .08 | -.06 [±.23] | 8.6 | .27 [-.26, .73] | 26.1 |
|  | -10 | .40 ± .08 | .03 [±.09] | 7.5 | .84 [.45, .96] | 23.7 | .97 ± .10 | -.09 [±.19]* | 7.6 | .47 [-.10, .83] | 23.7 |
|  | -15 | .42 ± .08 | .00 [±.13] | 10.2 | .70 [.14, .92] | 29.7 | .94 ± .15 | .09 [±.35] | 12.0 | .46 [-.12, .82] | 38.0 |
|  | -20 | .43 ± .11 | .04 [±.13] | 9.4 | .78 [.29, .95] | 33.4 | .94 ± .16 | -.14 [±.44] | 15.7 | .29 [-.25, .76] | 50.3 |
| WM Ventral | +20 | .47 ±.04 | -.01 [±.10] | 6.8 | .40 [-.32, .81] | 20.7 | .95 ± .12 | -.01 [±.14] | 4.5 | .84 [.47, .96] | 14.0 |
|  | +15 | .43 ± .06 | .00 [±.11] | 6.9 | .66 [.07, .91] | 23.0 | .95 ± .07 | -.06 [±.20] | 6.6 | .23 [-.26, .70] | 22.5 |
|  | +10 | .43 ± .07 | -.02 [±.11] | 6.5 | .77 [.33, .94] | 23.9 | .96 ± .08 | -.02 [±.14] | 4.4 | .66 [.11, .90] | 13.9 |
|  | +5 | .43 ± .07 | -.02 [±.06]* | 5.0 | .89 [.48, .97] | 15.6 | .91 ± .08 | -.05 [±.13] | 4.2 | .64 [.08, .90] | 15.8 |
|  | 0 | .42 ± .07 | -.01 [±.11] | 7.1 | .73 [.23, .92] | 23.9 | .88 ± .08 | -.05 [±.20] | 7.6 | .42 [-.15, .81] | 24.0 |
|  | -5 | .44 ± .07 | -.03 [±.10] | 6.5 | .73 [.27, .92] | 24.9 | .89 ± .04 | -.02 [±.17] | 5.6 | .05 [-.60, .64] | 18.5 |
|  | -10 | .42 ± .05 | -.01 [±.12] | 8.8 | .53 [-.13, .86] | 27.5 | .91 ± .08 | -.05 [±.22] | 6.4 | .27 [-.33, .74] | 24.8 |
|  | -15 | .37 ± .08 | -.01 [±.17] | 14.2 | .51 [-.18, .85] | 45.0 | .93 ± .11 | .00 [±.28] | 7.2 | .42 [-.32, .82] | 29.2 |
|  | -20 | .36 ± .10 | -.02 [±.15] | 13.8 | .75 [.24, .94] | 38.9 | .90 ± .14 | .02 [±.30] | 9.4 | .57 [-.15, .88] | 30.9 |

* Indicates significant difference between scan and rescan (p < 0.05).

*Notes:* The individual axial slice stacks were aligned at the LSE landmark, defined as the slice with the largest gray matter CSA ($\mathrm{GM}_{max,mw}$), without adjusting for the length of the conus medullaris. The landmarks were determined in the first scan. A positive distance indicates a rostral direction from the LSE landmark. For a single subject, DTI metrics were not available for slices with coordinates -20 mm (n=9).

*Abbreviations:* CI, confidence interval; CV, scan-rescan coefficient of variation; $\bar{d}$, mean scan-rescan difference; ICC, scan-rescan intraclass correlation coefficient; LSE, lumbosacral enlargement; MDC, minimal detectable change; SD, standard deviation; WM, white matter.
