# Supplementary material for: Improved inter-subject alignment of the lumbosacral cord for group-level in vivo gray and white matter assessments: A scan-rescan MRI study at 3T
Source: PLoS One. 2024 Apr 16;19(4):e0301449. doi: 10.1371/journal.pone.0301449 (PMC11020367; doi:10.1371/journal.pone.0301449)
Supplement: S6 Table — (DOCX) [file pone.0301449.s007.docx]

**S7 Table.** Slice-wise scan-rescan reliability of axial and radial diffusivity values (n=10 healthy volunteers).

|  | Distance from LSE landmark (mm) | **Axial Diffusivity** (10^-3^ mm^2^/s) | | | | | **Radial Diffusivity** (10^-3^ mm^2^/s) | | | | |
| --- | --- | --- | --- | --- | --- | --- | --- | --- | --- | --- | --- |
|  |  | mean ± SD | $\bar{d}$  [± 1.96 SD] | CV  (%) | ICC  [95% CI] | MDC  (%) | mean ± SD | $\bar{d}$  [± 1.96 SD] | CV  (%) | ICC  [95% CI] | MDC  (%) |
| Gray Matter | +20 | 1.34 ± .16 | .09 [±.18]* | 5.4 | .73 [.04, .93] | 17.6 | .66 ± .04 | .01 [±.08] | 3.7 | .64 [.10, .90] | 12.1 |
|  | +15 | 1.27 ± .13 | .01 [±.18] | 4.2 | .79 [.35, .94] | 13.2 | .68 ± .04 | -.01 [±.22] | 7.5 | -.45 [-.97, .30] | 32.4 |
|  | +10 | 1.21 ± .07 | .01 [±.14] | 3.2 | .67 [.10, .91] | 10.7 | .70 ± .06 | .02 [±.11] | 4.1 | .63 [.07, .89] | 15.8 |
|  | +5 | 1.15 ± .07 | -.02 [±.13] | 3.3 | .62 [.05, .89] | 10.7 | .67 ± .05 | .01 [±.09] | 3.6 | .65 [.09, .90] | 12.7 |
|  | 0 | 1.11 ± .10 | .00 [±.23] | 5.7 | .49 [-.21, .85] | 19.6 | .65 ± .06 | -.01 [±.15] | 6.5 | .52 [-.14, .86] | 21.5 |
|  | -5 | 1.09 ± .09 | .00 [±.26] | 6.7 | .32 [-.43, .78] | 23.2 | .65 ± .04 | .02 [±.14] | 7.0 | .17 [-.49, .70] | 21.7 |
|  | -10 | 1.10 ± .09 | -.01 [±.21] | 5.3 | .53 [-.16, .86] | 18.3 | .68 ± .04 | -.01 [±.17] | 7.4 | -.18 [-.82, .51] | 24.0 |
|  | -15 | 1.07 ± .11 | -.01 [±.17] | 4.3 | .72 [.20, .92] | 15.5 | .67 ± .05 | -.01 [±.11] | 4.8 | .44 [-.22, .82] | 16.4 |
|  | -20 | 1.12 ± .13 | .01 [±.29] | 7.2 | .51 [-.24, .87] | 24.8 | .66 ± .08 | .00 [±.20] | 6.8 | .38 [-.42, .82] | 29.4 |
| White Matter | +20 | 1.66 ± .19 | .00 [±.12] | 2.1 | .96 [.84, .99] | 6.6 | .63 ± .06 | .00 [±.11] | 4.6 | .70 [.13, .92] | 16.5 |
|  | +15 | 1.60 ± .16 | -.05 [±.19] | 3.7 | .81 [.41, .95] | 12.6 | .65 ± .04 | -.02 [±.22] | 7.4 | -.41, [-.92, .32] | 33.3 |
|  | +10 | 1.57 ± .11 | -.02 [±.15] | 3.2 | .79 [.38, .94] | 9.3 | .66 ± .07 | .00 [±.12] | 5.6 | .68 [.11, .91] | 17.6 |
|  | +5 | 1.51 ± .14 | -.07 [±.15]* | 3.6 | .78 [.18, .95] | 12.6 | .68 ± .07 | -.05 [±.16] | 8.2 | .39 [-.14, .79] | 25.9 |
|  | 0 | 1.45 ± .15 | -.02 [±.26] | 4.8 | .69 [.14, .91] | 16.8 | .64 ± .07 | -.04 [±.16] | 7.6 | .48 [-.09, .83] | 25.3 |
|  | -5 | 1.42 ± .16 | -.03 [±.24] | 5.3 | .75 [.29, .93] | 16.4 | .64 ± .05 | -.01 [±.13] | 6.1 | .36 [-.36, .80] | 19.7 |
|  | -10 | 1.44 ± .20 | -.06 [±.30] | 6.0 | .74 [.29, .93] | 21.1 | .70 ± .08 | -.04 [±.18] | 6.9 | .46 [-.12, .83] | 26.3 |
|  | -15 | 1.37 ± .16 | .03 [±.26] | 5.4 | .72 [.23, .92] | 18.2 | .70 ± .08 | .04 [±.22] | 8.8 | .32 [-.29, .77] | 32.3 |
|  | -20 | 1.37 ± .20 | -.05 [±.40] | 8.4 | .61 [-.05, .90] | 27.6 | .70 ± .11 | -.08 [±.24] | 11.7 | .47 [-.11, .84] | 37.7 |
| WM Dorsal | +20 | 1.84 ± .21 | .04 [±.25] | 4.0 | .83 [.48, .95] | 13.4 | .59 ± .10 | .02 [±.25] | 10.0 | .38 [-.33, .80] | 41.7 |
|  | +15 | 1.77 ± .18 | -.02 [±.30] | 4.9 | .71 [.18, .92] | 16.0 | .61 ± .07 | .01 [±.25] | 10.7 | .06 [-.66, .66] | 40.3 |
|  | +10 | 1.70 ± .13 | .02 [±.21] | 3.7 | .72 [.21, .92] | 11.9 | .59 ± .09 | .01 [±.19] | 9.6 | .54 [-.14, .87] | 30.5 |
|  | +5 | 1.63 ± .13 | .04 [±.26] | 4.2 | .59 [.01, .88] | 15.4 | .62 ± .08 | .00 [±.22] | 8.7 | .42 [-.32, .82] | 33.6 |
|  | 0 | 1.57 ± .12 | .08 [±.27] | 5.1 | .43 [-.12, .81] | 18.3 | .59 ± .09 | .01 [±.13] | 6.2 | .77 [.31, .94] | 21.1 |
|  | -5 | 1.51 ± .20 | .06 [±.25] | 5.8 | .78 [.37, .94] | 17.6 | .59 ± .08 | .04 [±.15] | 8.4 | .55 [.00, .86] | 27.2 |
|  | -10 | 1.50 ± .33 | .00 [±.36] | 7.2 | .87 [.55, .97] | 22.5 | .68 ± .20 | .00 [±.24] | 10.6 | .84 [.47, .96] | 32.9 |
|  | -15 | 1.39 ± .17 | -.01 [±.37] | 7.7 | .55 [-.12, .87] | 24.9 | .65 ± .06 | .04 [±.22] | 10.2 | .13 [-.47, .67] | 34.1 |
|  | -20 | 1.43 ± .18 | .01 [±.43] | 9.1 | .49 [-.28, .86] | 28.5 | .68 ± .07 | -.10 [±.23]* | 13.9 | .14 [-.25, .64] | 39.9 |
| WM Lateral | +20 | 1.60 ± .16 | -.02 [±.18] | 3.2 | .86 [.53, .96] | 11.0 | .63 ± .05 | -.01 [±.12] | 5.7 | .52 [-.12, .85] | 18.4 |
|  | +15 | 1.55 ± .20 | -.04 [±.22] | 4.1 | .81 [.45, .95] | 14.3 | .65 ± .06 | -.04 [±.27] | 9.4 | -.15 [-.73, .52] | 42.1 |
|  | +10 | 1.54 ± .14 | -.04 [±.16] | 3.5 | .82 [.46, .95] | 10.7 | .68 ± .08 | -.02 [±.16] | 7.0 | .58 [-.04, .88] | 22.7 |
|  | +5 | 1.53 ± .16 | -.16 [±.27]* | 7.5 | .50 [-.11, .85] | 24.7 | .74 ± .10 | -.13 [±.29]* | 12.6 | .18 [-.21, .64] | 46.7 |
|  | 0 | 1.44 ± .19 | -.06 [±.31] | 6.7 | .70 [.21, .92] | 21.7 | .67 ± .10 | -.09 [±.29] | 13.4 | .29 [-.23, .74] | 46.2 |
|  | -5 | 1.40 ± .18 | -.08 [±.34] | 7.7 | .58 [.03, .87] | 25.0 | .67 ± .06 | -.06 [±.18] | 9.6 | .30 [-.21, .74] | 28.5 |
|  | -10 | 1.44 ± .21 | -.11 [±.30] | 6.7 | .69 [.15, .91] | 23.5 | .74 ± .08 | -.08 [±.16]* | 8.5 | .41 [-.12, .80] | 26.8 |
|  | -15 | 1.39 ± .24 | .11 [±.40] | 10.5 | .65 [.12, .90] | 30.2 | .71 ± .14 | .07 [±.35] | 15.0 | .39 [-.20, .80] | 49.6 |
|  | -20 | 1.40 ± .24 | -.12 [±.65] | 13.6 | .36 [-.32, .80] | 46.0 | .71 ± .16 | -.15 [±.37]* | 19.1 | .39 [-.15, .80] | 59.1 |
| WM Ventral | +20 | 1.51 ±. 23 | -.02 [±.19] | 3.6 | .92 [.72, .98] | 12.0 | .67 ± .07 | .00 [±.14] | 5.8 | .55 [-.13, .87] | 20.3 |
|  | +15 | 1.45 ±. 17 | -.10 [±.28]* | 6.5 | .60 [.03, .88] | 22.2 | .70 ± .05 | -.04 [±.18] | 7.9 | -.03 [-.53, .55] | 27.1 |
|  | +10 | 1.46 ±. 15 | -.06 [±.20] | 4.6 | .75 [.29, .93] | 14.5 | .71 ± .09 | .00 [±.15] | 6.4 | .70 [.14, .92] | 19.8 |
|  | +5 | 1.37 ±. 17 | -.08 [±.20]* | 4.1 | .76 [.23, .94] | 17.0 | .68 ± .07 | -.03 [±.11] | 5.4 | .72 [.24, .92] | 16.7 |
|  | 0 | 1.34 ±. 18 | -.09 [±.33] | 8.2 | .60 [.05, .88] | 25.8 | .65 ± .06 | -.03 [±.17] | 8.4 | .32 [-.29, .77] | 27.0 |
|  | -5 | 1.36 ±. 14 | -.06 [±.26] | 6.4 | .61 [.07, .88] | 19.8 | .65 ± .04 | .00 [±.15] | 5.9 | .07 [-.66, .67] | 22.2 |
|  | -10 | 1.37 ±. 17 | -.07 [±.28] | 6.5 | .67 [.16, .90] | 21.3 | .68 ± .05 | -.03 [±.21] | 8.2 | -.10 [-.67, .54] | 31.0 |
|  | -15 | 1.33 ±. 19 | -.02 [±.49] | 8.9 | .41 [-.32, .82] | 35.2 | .73 ± .09 | .00 [±.23] | 8.1 | .43 [-.29, .83] | 30.6 |
|  | -20 | 1.27 ±. 23 | .00 [±.34] | 6.9 | .76 [.23, .94] | 25.0 | .72 ± .12 | .02 [±.30] | 12.6 | .48 [-.27, .85] | 39.7 |

* Indicates significant difference between scan and rescan (p < 0.05).

*Notes:* The individual axial slice stacks were aligned at the LSE landmark, defined as the slice with the largest gray matter CSA ($\mathrm{GM}_{max,mw}$), without adjusting for the length of the conus medullaris. The landmarks were determined in the first scan. A positive distance indicates a rostral direction from the LSE landmark. For a single subject, DTI metrics were not available for slices with coordinates -20 mm (n=9).

*Abbreviations:* CI, confidence interval; CV, scan-rescan coefficient of variation; $\bar{d}$, mean scan-rescan difference; ICC, scan-rescan intraclass correlation coefficient; LSE, lumbosacral enlargement; MDC, minimal detectable change; SD, standard deviation; WM, white matter.
