# Supplementary material for: Improved inter-subject alignment of the lumbosacral cord for group-level in vivo gray and white matter assessments: A scan-rescan MRI study at 3T
Source: PLoS One. 2024 Apr 16;19(4):e0301449. doi: 10.1371/journal.pone.0301449 (PMC11020367; doi:10.1371/journal.pone.0301449)
Supplement: S7 Table — (DOCX) [file pone.0301449.s008.docx]

**S8 Table.** Scan-rescan reliability of cross-sectional area measurements (n=10 healthy volunteers).

|  | Segment | CSA (mm^2^)  mean ± SD | $\bar{d}$ (mm^2^)  [± 1.96 SD] | CV  (%) | ICC  [95% CI] | MDC  (%) |
| --- | --- | --- | --- | --- | --- | --- |
| Spinal Cord | +3 | 54.9 ± 5.5 | -0.2 [±5.0] | 2.5 | .91 [.67, .98] | 8.5 |
|  | +2 | 59.8 ± 5.5 | -0.8 [±6.7] | 2.5 | .83 [.47, .95] | 10.7 |
|  | +1 | 62.0 ± 5.7 | -0.0 [±2.4] | 1.1 | .98 [.92, .99] | 3.6 |
|  | LSE | 60.2 ± 6.2 | 0.3 [±4.1] | 2.1 | .95 [.81, .99] | 6.4 |
|  | -1 | 51.2 ± 6.0 | 0.8 [±7.8] | 3.9 | .81 [.42, .95] | 14.6 |
|  | -2 | 33.8 ± 5.5 | 0.9 [±6.9] | 6.2 | .82 [.45, .95] | 19.6 |
|  | -3 | 18.6 ± 2.9 | 0.1 [±6.9] | 10.9 | .48 [-.23, .84] | 35.3 |
|  | -4 | 9.5 ± 1.4 | 0.7 [±4.9] | 15.7 | .10 [-.54, .66] | 50.8 |
|  | -5 | 5.9 ± 1.6 | 0.2 [±2.6] | 11.4 | .72 [.20, .92] | 41.1 |
| Gray Matter | +3 | 17.7 ± 2.8 | 0.6 [±5.9] | 6.9 | .57 [-.05, .87] | 31.9 |
|  | +2 | 21.7 ± 2.8 | -0.4 [±5.5] | 6.2 | .62 [.01, .89] | 24.1 |
|  | +1 | 24.7 ± 3.2 | -0.2 [±3.5] | 3.7 | .86 [.53, .96] | 13.4 |
|  | LSE | 27.3 ± 3.7 | 0.4 [±2.8] | 2.9 | .93 [.76, .98] | 9.8 |
|  | -1 | 23.9 ± 3.5 | 1.2 [±3.1]* | 4.3 | .86 [.45, .97] | 15.2 |
|  | -2 | 15.7 ± 3.0 | 0.3 [±3.3] | 6.6 | .86 [.55, .96] | 19.9 |
|  | -3 | 8.4 ± 1.6 | -0.1 [±2.6] | 8.6 | .71 [.16, .92] | 29.5 |
|  | -4 | 3.8 ± 0.6 | 0.2 [±1.9] | 12.8 | .14 [-.55, .70] | 50.7 |
|  | -5 | 1.9 ± 0.6 | -0.1 [±1.4] | 18.5 | .55 [-.13, .87] | 69.5 |
| White Matter | +3 | 37.2 ± 3.1 | -0.9 [±5.8] | 4.4 | .64 [.08, .89] | 15.3 |
|  | +2 | 38.2 ± 3.1 | -0.4 [±2.3] | 1.9 | .93 [.75, .98] | 5.9 |
|  | +1 | 37.3 ± 3.1 | 0.2 [±1.9] | 1.4 | .96 [.84, .99] | 4.9 |
|  | LSE | 32.9 ± 3.1 | -0.1 [±5.4] | 4.4 | .70 [.14, .92] | 15.5 |
|  | -1 | 27.3 ± 2.8 | -0.4 [±5.7] | 5.3 | .60 [-.03, .88] | 19.8 |
|  | -2 | 18.0 ± 2.7 | 0.6 [±4.1] | 6.0 | .75 [.28, .93] | 21.9 |
|  | -3 | 10.3 ± 1.4 | 0.2 [±4.4] | 12.7 | .20 [-.54, .73] | 41.5 |
|  | -4 | 5.8 ± 0.9 | 0.5 [±3.2] | 17.6 | .10 [-.52, .66] | 54.7 |
|  | -5 | 4.0 ± 1.1 | 0.3 [±1.6] | 11.2 | .74 [.28, .93] | 39.8 |

* Indicates significant difference between scan and rescan (p < 0.05).

*Notes:* The individual axial slice stacks were aligned at the LSE landmark, defined as the slice with the largest gray matter CSA ($\mathrm{GM}_{max,mw}$), and were adjusted for the length of the conus medullaris. The landmarks were determined independently for scan and rescan. A positive segment indicates a rostral direction from the LSE landmark.

*Abbreviations:* CI, confidence interval; CV, scan-rescan coefficient of variation; CSA, cross-sectional area; $\bar{d}$, mean scan-rescan difference; ICC, scan-rescan intraclass correlation coefficient; LSE, lumbosacral enlargement; MDC, minimal detectable change; SD, standard deviation.
