# Supplementary material for: Improved inter-subject alignment of the lumbosacral cord for group-level in vivo gray and white matter assessments: A scan-rescan MRI study at 3T
Source: PLoS One. 2024 Apr 16;19(4):e0301449. doi: 10.1371/journal.pone.0301449 (PMC11020367; doi:10.1371/journal.pone.0301449)
Supplement: S8 Table — (DOCX) [file pone.0301449.s009.docx]

**S9 Table.** Scan-rescan reliability of fractional anisotropy and mean diffusivity values (n=10 healthy volunteers).

|  |  | **Fractional Anisotropy** | | | | | **Mean Diffusivity** (10^-3^ mm^2^/s) | | | | |
| --- | --- | --- | --- | --- | --- | --- | --- | --- | --- | --- | --- |
|  | Segment | mean ± SD | $\bar{d}$  [± 1.96 SD] | CV  (%) | ICC  [±95% CI] | MDC  (%) | mean ± SD | $\bar{d}$  [± 1.96 SD] | CV  (%) | ICC  [95% CI] | MDC  (%) |
| Gray Matter | +3 | .46 ± .06 | -.01 [±.10] | 6.0 | .74 [.21, .94] | 20.0 | .91 ± .04 | .01 [±.09] | 3.0 | .46 [-.25, .85] | 9.7 |
|  | +2 | .39 ± .06 | .01 [±.12] | 8.1 | .60 [-.03, .88] | 28.2 | .87 ± .05 | .01 [±.13] | 4.5 | .36 [-.36, .80] | 14.8 |
|  | +1 | .34 ± .05 | -.01 [±.10] | 9.8 | .64 [.04, .90] | 28.7 | .85 ± .04 | .02 [±.09] | 3.1 | .44 [-.17, .82] | 10.2 |
|  | LSE | .33 ± .04 | .01 [±.07] | 6.3 | .70 [.17, .92] | 21.1 | .81 ± .04 | -.01 [±.15] | 5.5 | .13 [-.58, .69] | 18.4 |
|  | -1 | .32 ± .06 | -.01 [±.04] | 4.8 | .92 [.69, .98] | 14.7 | .81 ± .04 | .00 [±.14] | 5.4 | .01 [-.70, .63] | 16.8 |
|  | -2 | .30 ± .06 | .00 [±.09] | 6.5 | .77 [.29, .94] | 28.2 | .82 ± .03 | -.02 [±.11] | 3.8 | .17 [-.40, .69] | 13.4 |
|  | -3 | .30 ± .09 | -.04 [±.13] | 15.2 | .74 [.15, .95] | 45.4 | .81 ± .08 | .00 [±.22] | 7.1 | .34 [-.66, .85] | 25.1 |
| White Matter | +3 | .58 ± .03 | -.02 [±.10] | 5.1 | .05 [-.61, .66] | 16.8 | 1.01 ± .07 | .00 [±.10] | 3.0 | .80 [.31, .95] | 9.1 |
|  | +2 | .53 ± .04 | -.01 [±.12] | 6.4 | .31 [-.40, .77] | 20.9 | .98 ± .08 | -.02 [±.14] | 3.7 | .66 [.10, .90] | 14.0 |
|  | +1 | .50 ± .06 | -.01 [±.08] | 5.1 | .78 [.37, .94] | 16.5 | .94 ± .06 | .01 [±.10] | 2.9 | .64 [.04, .90] | 10.6 |
|  | LSE | .47 ± .06 | .01 [±.06] | 3.8 | .87 [.58, .97] | 12.8 | .92 ± .07 | -.03 [±.17] | 5.6 | .43 [-.19, .82] | 18.2 |
|  | -1 | .44 ± .06 | -.01 [±.06] | 4.8 | .89 [.62, .97] | 13.3 | .90 ± .07 | -.01 [±.22] | 7.4 | .30 [-.44, .77] | 23.3 |
|  | -2 | .40 ± .06 | .00 [±.09] | 5.8 | .79 [.34, .94] | 21.3 | .92 ± .05 | -.03 [±.19] | 6.0 | .07 [-.57, .65] | 20.4 |
|  | -3 | .36 ± .07 | -.01 [±.14] | 11.5 | .65 [-.14, .93] | 35.0 | .94 ± .12 | -.08 [±.22] | 8.6 | .56 [-.10, .90] | 26.2 |
| WM Dorsal | +3 | .65 ± .02 | -.03 [±.13] | 6.7 | -.33 [-.76, .37] | 20.1 | 1.04 ± .09 | .02 [±.14] | 4.2 | .74 [.25, .93] | 12.8 |
|  | +2 | .62 ± .05 | -.02 [±.13] | 6.3 | .46 [-.20, .83] | 20.5 | 1.01 ± .09 | .01 [±.16] | 3.8 | .69 [.13, .91] | 14.6 |
|  | +1 | .59 ± .07 | -.02 [±.11] | 5.7 | .71 [.21, .92] | 18.6 | .94 ± .06 | .04 [±.13] | 4.6 | .40 [-.14, .79] | 15.1 |
|  | LSE | .56 ± .07 | .01 [±.09] | 4.6 | .81 [.42, .95] | 15.4 | .90 ± .07 | .01 [±.19] | 5.6 | .41 [-.30, .82] | 20.6 |
|  | -1 | .51 ± .07 | -.01 [±.08] | 5.0 | .87 [.56, .96] | 15.0 | .86 ± .08 | .03 [±.19] | 5.3 | .51 [-.12, .85] | 21.6 |
|  | -2 | .45 ± .08 | -.01 [±.11] | 6.9 | .81 [.40, .95] | 22.1 | .86 ± .05 | -.03 [±.18] | 4.7 | .01 [-.61, .61] | 21.3 |
|  | -3 | .40 ± .08 | .03 [±.12] | 8.5 | .71 [.09, .94] | 29.7 | .94 ± .12 | -.05 [±.40] | 8.8 | .14 [-.70, .78] | 41.6 |
| WM Lateral | +3 | .57 ± .04 | -.01 [±.09] | 4.2 | .58 [-.08, .89] | 14.5 | .99 ± .07 | -.03 [±.13] | 4.2 | .60 [.01, .89] | 13.1 |
|  | +2 | .53 ± .04 | .00 [±.12] | 6.1 | .29 [-46, .77] | 21.8 | .97 ± .09 | -.04 [±.19] | 5.3 | .55 [-.03, .86] | 19.7 |
|  | +1 | .49 ± .06 | -.01 [±.11] | 6.7 | .63 [.03, .90] | 22.0 | .95 ± .10 | -.02 [±.17] | 5.7 | .70 [.20, .92] | 17.1 |
|  | LSE | .45 ± .06 | .02 [±.09] | 6.4 | .69 [.19, .91] | 21.3 | .95 ± .12 | -.07 [±.23] | 8.0 | .55 [-.01, .86] | 25.8 |
|  | -1 | .41 ± .07 | .01 [±.08] | 5.7 | .86 [.57, .96] | 18.6 | .97 ± .11 | -.05 [±.40] | 12.8 | .10 [-.57, .67] | 40.0 |
|  | -2 | .39 ± .07 | .02 [±.13] | 10.2 | .66 [.12, .90] | 32.4 | .98 ± .09 | -.04 [±.37] | 10.6 | -.03 [-.68, .60] | 36.9 |
|  | -3 | .37 ± .07 | -.01 [±.17] | 13.2 | .47 [-.48, .89] | 44.1 | .97 ± .15 | -.18 [±.33]* | 14.7 | .36 [-.17, .82] | 43.6 |
| WM Ventral | +3 | .51 ± .04 | -.01 [±.11] | 6.6 | .27 [-.52, .78] | 21.1 | .99 ± .09 | .00 [±.18] | 4.5 | .61 [-.10, .90] | 16.7 |
|  | +2 | .45 ± .05 | -.02 [±.13] | 8.3 | .47 [-.19, .84] | 27.5 | .95 ± .09 | -.02 [±.13] | 3.7 | .74 [.28, .93] | 13.4 |
|  | +1 | .43 ± .07 | -.02 [±.07] | 5.4 | .85 [.48, .96] | 17.4 | .92 ± .05 | .01 [±.13] | 3.4 | .46 [-.24, .84] | 13.7 |
|  | LSE | .41 ± .06 | -.01 [±.07] | 5.1 | .84 [.49, .96] | 16.1 | .88 ± .05 | -.02 [±.20] | 6.5 | -.07 [-.71, .58] | 22.2 |
|  | -1 | .41 ± .05 | -.03 [±.10] | 7.2 | .60 [.06, .88] | 24.7 | .86 ± .05 | -.01 [±.17] | 6.2 | .21 [-.51, .73] | 19.1 |
|  | -2 | .37 ± .06 | -.01 [±.12] | 9.0 | .56 [-.08, .87] | 32.5 | .90 ± .07 | -.01 [±.15] | 4.9 | .58 [-.05, .88] | 15.7 |
|  | -3 | .31 ± .09 | -.05 [±.14] | 16.9 | .64 [-.01, .93] | 51.6 | .91 ± .13 | .01 [±.28] | 7.5 | .54 [-.37, .91] | 29.0 |

* Indicates significant difference between scan and rescan (p < 0.05).

*Notes:* The individual axial slice stacks were aligned at the LSE landmark, defined as the slice with the largest gray matter CSA ($\mathrm{GM}_{max,mw}$), and were adjusted for the length of the conus medullaris. The landmarks were determined independently for scan and rescan. A positive segment indicates a rostral direction from the LSE landmark. DTI metrics were not available for segment LSE+3 in one subject (n=9) and for segment LSE-3 in three subjects (n=7).

*Abbreviation*s: CI, confidence interval; CV, scan-rescan coefficient of variation; $\bar{d}$, mean scan-rescan difference; ICC, scan-rescan intraclass correlation coefficient; LSE, lumbosacral enlargement; MDC, minimal detectable change; SD, standard deviation; WM, white matter.
