# Supplementary material for: Improved inter-subject alignment of the lumbosacral cord for group-level in vivo gray and white matter assessments: A scan-rescan MRI study at 3T
Source: PLoS One. 2024 Apr 16;19(4):e0301449. doi: 10.1371/journal.pone.0301449 (PMC11020367; doi:10.1371/journal.pone.0301449)
Supplement: S9 Table — (DOCX) [file pone.0301449.s010.docx]

**S10 Table**. Scan-rescan reliability of axial and radial diffusivity values (n=10 healthy volunteers).

|  |  | **Axial Diffusivity** (10^-3^ mm^2^/s) | | | | | **Radial Diffusivity** (10^-3^ mm^2^/s) | | | | |
| --- | --- | --- | --- | --- | --- | --- | --- | --- | --- | --- | --- |
|  | Segment | mean ± SD | $\bar{d}$  [± 1.96 SD] | CV  (%) | ICC  [95% CI] | MDC  (%) | mean ± SD | $\bar{d}$  [± 1.96 SD] | CV  (%) | ICC  [95% CI] | MDC  (%) |
| Gray Matter | +3 | 1.43 ± .14 | .00 [±.24] | 5.2 | .70 [.08, .92] | 15.4 | .64 ± .04 | .02 [±.07] | 3.8 | .53 [-.09, .87] | 11.6 |
|  | +2 | 1.29 ± .13 | .03 [±.21] | 4.5 | .73 [.24, .92] | 15.6 | .66 ± .04 | .00 [±.15] | 5.0 | -.01 [-.72, .62] | 21.9 |
|  | +1 | 1.19 ± .08 | .02 [±.17] | 4.3 | .51 [-.13, .85] | 14.0 | .68 ± .05 | .01 [±.10] | 3.9 | .60 [.01, .88] | 14.3 |
|  | LSE | 1.11 ± .09 | -.01 [±.18] | 4.7 | .62 [-.01, .89] | 15.5 | .66 ± .04 | -.02 [±.14] | 6.4 | .04 [-.63, .64] | 21.6 |
|  | -1 | 1.10 ± .09 | .00 [±.20] | 5.2 | .57 [-.09, .88] | 16.8 | .66 ± .04 | .01 [±.12] | 5.7 | .27 [-.46, .76] | 16.9 |
|  | -2 | 1.10 ± .09 | -.02 [±.19] | 4.8 | .59 [-.02, .88] | 16.4 | .68 ± .03 | -.02 [±.10] | 4.4 | .28 [-.27, .74] | 14.8 |
|  | -3 | 1.08 ± .16 | -.06 [±.31] | 7.5 | .59 [-.15, .91] | 27.9 | .67 ± .07 | .03 [±.20] | 7.9 | .38 [-.48, .86] | 28.5 |
| White Matter | +3 | 1.76 ± .16 | -.02 [±.21] | 3.0 | .81 [.39, .95] | 11.0 | .63 ± .03 | .01 [±.12] | 5.7 | .13 [-.62, .72] | 18.1 |
|  | +2 | 1.65 ± .18 | -.04 [±.19] | 4.1 | .85 [.54, .96] | 11.5 | .64 ± .04 | -.01 [±.17] | 6.1 | .02 [-.69, .64] | 25.2 |
|  | +1 | 1.53 ± .14 | .00 [±.16] | 3.2 | .85 [.51, .96] | 9.6 | .64 ± .06 | .01 [±.12] | 4.7 | .60 [-.01, .88] | 17.7 |
|  | LSE | 1.44 ± .14 | -.02 [±.22] | 3.9 | .74 [.26, .93] | 14.3 | .65 ± .06 | -.03 [±.15] | 7.7 | .43 [-.17, .81] | 23.7 |
|  | -1 | 1.38 ± .15 | -.02 [±.33] | 7.0 | .54 [-.12, .87] | 22.6 | .67 ± .07 | -.01 [±.18] | 7.8 | .39 [-.34, .81] | 25.3 |
|  | -2 | 1.34 ± .12 | -.03 [±.29] | 6.1 | .45 [-.22, .83] | 20.7 | .71 ± .05 | -.02 [±.16] | 6.4 | .26 [-.39, .74] | 21.9 |
|  | -3 | 1.33 ± .20 | -.13 [±.29] | 6.9 | .67 [-.01, .93] | 26.1 | .75 ± .10 | -.06 [±.22] | 10.2 | .51 [-.18, .89] | 29.7 |
| WM Dorsal | +3 | 1.94 ± .17 | -.01 [±.22] | 3.1 | .81 [.34, .95] | 10.8 | .59 ± .06 | .04 [±.19] | 10.4 | .24 [-.37, .75] | 31.8 |
|  | +2 | 1.84 ± .18 | -.01 [±.27] | 4.7 | .76 [.28, .94] | 13.7 | .60 ± .08 | .02 [±.18] | 7.7 | .56 [-.09, .87] | 29.0 |
|  | +1 | 1.68 ± .14 | .05 [±.20] | 3.7 | .73 [.27, .92] | 12.3 | .57 ± .08 | .04 [±.16] | 8.0 | .54 [-.02, .86] | 28.4 |
|  | LSE | 1.55 ± .12 | .04 [±.24] | 4.4 | .59 [.01, .88] | 15.2 | .58 ± .09 | .00 [±.18] | 8.3 | .61 [-.03, .89] | 29.9 |
|  | -1 | 1.42 ± .17 | .03 [±.29] | 4.8 | .68 [.14, .91] | 19.8 | .59 ± .08 | .03 [±.16] | 7.6 | .60 [.02, .88] | 26.1 |
|  | -2 | 1.32 ± .13 | -.05 [±.21] | 3.9 | .69 [.20, .91] | 16.4 | .62 ± .05 | -.02 [±.18] | 6.2 | .17 [-.54, .71] | 28.5 |
|  | -3 | 1.38 ± .20 | -.03 [±.48] | 6.7 | .48 [-.45, .89] | 32.4 | .72 ± .10 | -.07 [±.38] | 13.1 | .08 [-.69, .75] | 52.1 |
| WM Lateral | +3 | 1.71 ± .16 | -.06 [±.22] | 3.8 | .76 [.29, .94] | 13.3 | .63 ± .04 | -.02 [±.14] | 6.6 | .20 [-.54, .74] | 21.3 |
|  | +2 | 1.62 ± .18 | -.05 [±.22] | 4.8 | .82 [.46, .95] | 13.7 | .65 ± .06 | -.03 [±.21] | 7.8 | .05 [-.59, .63] | 31.8 |
|  | +1 | 1.52 ± .18 | -.04 [±.23] | 4.8 | .81 [.43, .95] | 14.6 | .67 ± .09 | -.02 [±.17] | 8.3 | .60 [-.01, .88] | 24.8 |
|  | LSE | 1.45 ± .20 | -.07 [±.28] | 6.1 | .75 [.29, .93] | 20.0 | .71 ± .09 | -.07 [±.22] | 10.3 | .39 [-.15, .79] | 34.1 |
|  | -1 | 1.43 ± .19 | -.04 [±.55] | 11.3 | .32 [-.40, .78] | 37.0 | .74 ± .10 | -.05 [±.33] | 14.2 | .17 [-.49, .70] | 44.2 |
|  | -2 | 1.41 ± .18 | -.02 [±.57] | 11.7 | .22 [-.52, .74] | 39.2 | .77 ± .07 | -.05 [±.28] | 10.1 | .03 [-.57, .62] | 36.7 |
|  | -3 | 1.36 ± .23 | -.25 [±.48]* | 13.6 | .39 [-.17, .84] | 44.7 | .77 ± .13 | -.14 [±.30] | 15.7 | .38 [-.19, .84] | 46.8 |
| WM Ventral | +3 | 1.62 ± .18 | .00 [±.40] | 6.6 | .54 [-.20, .88] | 23.4 | .67 ± .05 | .00 [±.10] | 4.6 | .63 [-.04, .91] | 14.4 |
|  | +2 | 1.48 ± .20 | -.05 [±.23] | 5.3 | .83 [.49, .96] | 15.8 | .69 ± .04 | -.01 [±.14] | 5.6 | .22 [-.52, .74] | 19.4 |
|  | +1 | 1.39 ± .14 | -.02 [±.19] | 3.5 | .78 [.34, .94] | 13.2 | .68 ± .06 | .02 [±.12] | 5.1 | .57 [-.01, .87] | 17.8 |
|  | LSE | 1.32 ± .13 | -.03 [±.26] | 5.9 | .57 [-.05, .87] | 19.1 | .66 ± .05 | -.02 [±.18] | 7.5 | .01 [-.67, .63] | 26.4 |
|  | -1 | 1.27 ± .12 | -.04 [±.29] | 7.2 | .46 [-.18, .83] | 22.4 | .65 ± .04 | .00 [±.13] | 5.8 | .26 [-.49, .76] | 19.8 |
|  | -2 | 1.28 ± .12 | -.03 [±.29] | 6.8 | .49 [-.18, .84] | 21.4 | .71 ± .07 | .00 [±.11] | 4.8 | .72 [.19, .92] | 15.2 |
|  | -3 | 1.23 ± .22 | -.06 [±.27] | 4.7 | .82 [.34, .97] | 21.5 | .74 ± .11 | .05 [±.30] | 10.6 | .32 [-.53, .84] | 39.3 |

* Indicates significant difference between scan and rescan (p < 0.05).

*Notes:* The individual axial slice stacks were aligned at the LSE landmark, defined as the slice with the largest gray matter CSA ($\mathrm{GM}_{max,mw}$), and were adjusted for the length of the conus medullaris. The landmarks were determined independently for scan and rescan. A positive segment indicates a rostral direction from the LSE landmark. DTI metrics were not available for segment LSE+3 in one subject (n=9) and for segment LSE-3 in three subjects (n=7).

*Abbreviations:* CI, confidence interval; CV, scan-rescan coefficient of variation; $\bar{d}$, mean scan-rescan difference; ICC, scan-rescan intraclass correlation coefficient; LSE, lumbosacral enlargement; MDC, minimal detectable change; SD, standard deviation; WM, white matter.
