# Supplementary material for: Improved inter-subject alignment of the lumbosacral cord for group-level in vivo gray and white matter assessments: A scan-rescan MRI study at 3T
Source: PLoS One. 2024 Apr 16;19(4):e0301449. doi: 10.1371/journal.pone.0301449 (PMC11020367; doi:10.1371/journal.pone.0301449)
Supplement: Attachment — Submitted filename: PLOS_ONE_Peer_Review___Original_Article___PONE_D_23_37329.pdf [file pone.0301449.s011.pdf]

PLOS ONE

PONE-D-23-37329
Original Research Article

January 29, 2024

Thank you for the opportunity to review your manuscript. The paper entitled "*Improved inter-subject alignment of the lumbosacral cord for group-level in vivo gray and white matter assessments: A scan-rescan MRI study at 3T*" presents a significant study focused on improving inter-subject alignment of the lumbosacral cord for group-level in vivo gray and white matter assessments in MRI studies. This research is pivotal in advancing our understanding of MRI metrics in the lumbosacral spinal cord, particularly in the context of challenges posed by variability in conus medullaris size and the lack of established image-based landmarks.

The study's approach, involving both healthy volunteers and patients with spinal cord injury, provides comprehensive insights into the scan-rescan reliability of MRI-based cross-sectional area measurements and diffusion tensor imaging metrics. While the findings are highly specialized and cater to a specific aspect of MRI research, they hold significant implications for enhancing the accuracy and reliability of MRI assessments in clinical and research settings.

> **Guidelines:** Conciseness and accuracy in writing are perennial challenges in the realm of research. Researchers are tasked with the complex endeavor of succinctly presenting intricate methodologies, findings, and supplementary materials such as tables and figures, while ensuring a coherent flow. To aid in this process, structured guidelines have been developed, catering to both experimental and nonexperimental study designs. Notably, publishers often endorse adherence to specific reporting standards. The EQUATOR Network, an abbreviation for 'Enhancing the QUAlity and Transparency Of health Research,' stands out as a pivotal resource in this context. This international organization is dedicated to augmenting the reliability and value of the medical research literature. It achieves this by championing the principles of transparent and precise reporting in research studies [1]. Researchers are encouraged to utilize the EQUATOR Network's guidelines and tools, available at EQUATOR Network, to bolster the integrity and credibility of their research projects and publications.

Major Comments

In recognition of the valuable contributions your study has made to the field of MRI research, particularly in spinal cord imaging, I would like to commend the authors for their diligent and innovative work. The study notably advances our understanding of MRI assessments in the lumbosacral spinal cord, addressing significant challenges with practical implications for both clinical practice and further research.

Design and Objectives: The study presents an experimental design focusing on MRI metrics analysis in the lumbosacral spinal cord. It involves a mixture of healthy volunteers and patients with spinal cord injury to assess the reliability of MRI-based cross-sectional area (CSA) measurements and diffusion tensor imaging (DTI) metrics. The objective is to enhance the inter-subject alignment for group-level analysis in MRI studies, addressing significant challenges in this area.

Methodology: The methodology is comprehensive, involving axial T2*-weighted and diffusion MRI at 3T. The study assesses the reliability of spinal cord and gray matter-based landmarks for inter-subject alignment, along with intra- and inter-rater reliability of CSA measurements and DTI metrics.

1. *Innovative Approach:* The strength of the study lies in its innovative approach to a complex problem in MRI analysis. However, a more detailed discussion on how this approach can be generalized or applied in other contexts would additionally strengthen the manuscript.

2. *Sample Diversity:* The inclusion of both healthy individuals and patients with spinal cord injury adds depth to the study. However, the manuscript could benefit from a further discussion on the potential biases or limitations this sample diversity might introduce.

3. *Reliability Measures:* The study presents compelling reliability measures, hence outlining concepts for the requirements in a multi-centre design (e.g. sample size) would enhance the manuscript's rigor.

4. *Technical Limitations and Challenges:* Delving deeper into the technical challenges and limitations encountered during the study, especially concerning MRI techniques and measurements, would provide valuable insights for future research.

5. *Impact of Participant Characteristics:* Given a limited sample size, the study should consider how further individual differences in participants, such as age, gender, or specific severity of spinal cord injury, might influence the MRI metrics.

6. *Future Research Directions:* Finally, the study should outline further potential directions for future research, especially in exploring other methods of alignment and landmark identification in MRI studies, and their clinical appicability.

Minor Comments

With appreciation for the study's strengths, these points are provided with the intent of further enriching the publication. From this reviewer's perspective, they are not mandatory for publication but are proposed as enhancements that could potentially add depth and clarity to your already substantial work.

1. *Detailed Reporting of Technical Parameters and code of analysis:* While the study's methodology is robust, and data can be accessed via github, providing more detailed descriptions of the technical parameters used in MRI assessments (e.g., specific settings or protocols including the analysis code) would significantly enhance the replicability and comprehension of the methods.

2. *Consideration of Technical Variabilities:* Discussing the potential technical variabilities that might impact the MRI measurements, such as machine calibration or operator expertise, would provide a more comprehensive view of the challenges in achieving reliable MRI metrics.

3. *Suggestions for Future Research:* Further offering specific suggestions for future research directions, particularly in areas that were not fully explored in the current study, would be valuable for advancing the field.

4. *Recommendations for Clinical Practice:* Lastly, providing clear recommendations for clinical practice based on the study's findings, especially in terms of improving MRI accuracy in spinal cord assessments, would enhance its applicability.

References

[1] K. A. Gould. The EQUATOR network. *Dimensions of Critical Care Nursing*, 35(6):350, 2016.
